# Comparing Sensitivity and Specificity of Addenbrooke's Cognitive Examination-I, III and Mini-Addenbrooke's Cognitive Examination in Parkinson's Disease

**DOI:** 10.1155/2018/5932028

**Published:** 2018-10-02

**Authors:** Tivadar Lucza, Zsuzsanna Ascherman, Márton Kovács, Attila Makkos, Márk Harmat, Annamária Juhász, József Janszky, Sámuel Komoly, Norbert Kovács, Krisztina Dorn, Kázmér Karádi

**Affiliations:** ^1^Institute of Behavioral Sciences, University of Pécs, Pécs, Hungary; ^2^Department of Neurology, University of Pécs, Pécs, Hungary; ^3^Doctoral School of Clinical Neuroscience, University of Pécs, Pécs, Hungary; ^4^MTA-PTE Clinical Neuroscience MR Research Group, Pécs, Hungary; ^5^Pediatric Clinic, University of Pécs, Pécs, Hungary

## Abstract

**Background:**

Parkinson's disease (PD) is the second most common neurodegenerative disorder characterized by numerous motor and nonmotor symptoms. Neurocognitive disorders (NCD) are one of the most troublesome problems and their diagnosis is often challenging.

**Methods:**

We compared the sensitivity and specificity of several versions of Addenbrooke Cognitive Examination (ACE, ACE-III, and Mini-ACE) on 552 subjects with PD. Normal cognition, mild and major NCD were judged in accordance with the respective criteria of the Diagnostic and Statistical Manual of Mental Disorders 5^th^ edition. Subsequently, we applied the receiver operation characteristic (ROC) analysis in comparison of different education levels.

**Results:**

For subjects with education level 0–8 and 9–12 years, the ACE-III had the best discriminating capabilities for mild NCD (cut-off scores: 83.5 and 85.5 points, respectively), while Mini-ACE was the best for subjects having education > 12 years (cut-off score: 25.5 points). For detecting major NCD, ACE-III had the best diagnostic accuracy in all levels of education (cut-off scores: 70.5, 77.5, and 78.5 points for subjects having education level 0–8, 9–12, and >12 years, respectively).

**Conclusion:**

ACE-III and its nested version, the Mini-ACE, had the best screening abilities for detecting mild and major NCD in PD.

## 1. Introduction

Parkinson's disease (PD) is the second most common neurodegenerative disorder characterized by both motor and nonmotor symptoms [1]. Besides the well-known motor symptoms including bradykinesia, resting tremor, and rigidity, numerous nonmotor symptoms (NMS) exist [2]. Among the NMSs, fatigue, autonomic disturbances, sleep disorders, depression, apathy, behavioral disturbances, and cognitive impairments are the most frequent and troublesome [3, 4]. Although some cognitive deficits do exist in the early stages of the disease interfering executive functions, working memory, and visuospatial functioning [5, 6], more pronounced neurocognitive disorders (NCDs) may develop as the disease progresses [7, 8]. These neuropsychological deficits are partly related to the dysfunction of dopaminergic frontostriatal circuits [9, 10].

NCDs are characterized by a decline from a previously attained level of cognitive functioning. This decline should be both clinically relevant in manner and objectively measurable in order to establish the diagnosis. In the clinical practice, basically, two diagnostic frameworks exist for diagnosing NCDs in PD.

The Task Forces and Committees of the International Parkinson's Disease and Movement Disorders Society (IPDMDS) established the criteria for mild cognitive impairment in PD (PD-MCI) [11] and the Parkinson's disease dementia (PDD) [12]. Although PD-MCI might develop in the early stages of PD [13], it is more characteristic of later phases. PD-MCI is defined as a stage between dementia and normal cognitive functioning with subjective concern and objective decline of cognitive functioning, without major impairment in the everyday functions [11, 14]. In addition to executive dysfunction, there is an alteration of posterior cortical functions in PD-MCI [15]. Prevalence of PD-MCI is increased by age, disease duration, and disease severity [11, 14]. Diagnosing PD-MCI is an important issue because PD-MCI predicts the development of dementia [16]. The establishment of the diagnosis of the PD-MCI is based on abbreviated (Level-I) and comprehensive (Level-II) assessments [11]. In accordance with the IPDMDS Task Force Guidelines, in PD-MCI, the cognitive functions (memory, executive function) are 1 to 2 standard deviations below the average level. PDD has more severe cortical dysfunctions compared with PD-MCI [12, 15]. Cortical profile of PDD represents an impairment of memory and language skill, while subcortical profile has a decline in attention, executive and visuospatial functions [17, 18]. PDD usually develops several years after the onset of motor symptoms [12]. The prevalence of PDD is approximately 20–40% [19–21]. The IPDMDS Task Force Guidelines provides a guideline for a diagnostic procedure for determining PDD [19, 22]. The diagnostic procedure also consists of abbreviated (Level-I) and comprehensive (Level-II) assessment as it is shown in PD-MCI. Detecting PDD is extremely important in the treatment of PD and in screening appropriate candidates for deep brain stimulation.

The fifth edition of the Diagnostic and Statistical Manual of Mental Disorders (DSM-5) also provides a common framework for the diagnosis of neurocognitive disorders, first by describing the main cognitive syndromes and then defining criteria to delineate specific etiological subtypes. The DSM-5 defines the mild and major NCDs in PD, which largely overlap with the PD-MCI and PDD terminology.

Diagnosing dementia in Parkinson's disease patient population poses significant challenges. The screening procedure includes several tests for the abbreviated assessment of PDD. These screening tools may represent short (Mini-Mental State Examination (MMSE) and Montreal Cognitive Examination [23] (MoCA)) and “longer” test batteries (e.g., Mattis Dementia Rating Scale and the Addenbrooke's Cognitive Examination) [19, 22, 24, 25].

The Addenbrooke's Cognitive Examination (ACE), a screening tool, has had substantial and rapid development over the years. The original version of ACE (ACE-I) was developed to differentiate Alzheimer's disease from frontotemporal dementia and to detect the early stages of these dementia syndromes [26]. The maximum score of ACE-I is 100, and higher numbers represent better functioning. ACE-I assesses six cognitive domains, where the maximum scores are 10 points for orientation, 8 points for attention, 35 points for memory, 14 points for verbal fluency, 28 points for language skills, and 5 points for visuospatial ability. ACE-I also includes the nested version of MMSE. The Spanish version of ACE-I was able to detect dementia in PD, and it could discriminate PDD from Alzheimer's dementia but not from frontotemporal dementia [27].

The Addenbrooke's Cognitive Examination-Revised (ACE-R) was developed soon after the publication of ACE-I considering the strengths and weaknesses of ACE-I [28] and had been translated into many languages [29–33]. While ACE-I has only one version, the ACE-R includes three alternative versions, allowing multiple testing over extended time. Significant changes were made in the language and visuospatial parts. ACE-R has five subscores representing various cognitive domains: attention/orientation (18 points), memory (26 points), verbal fluency (14 points), language skills (26 points), and visuospatial abilities (16 points). The maximum score is 100 in ACE-R. Recently, ACE-R has been used extensively worldwide [29, 32–34].

The newest version of the ACE test family is the Addenbrooke's Cognitive Examination-III (ACE-III) [35]. Its maximum score is also 100 points. Compared to ACE-R, ACE-III has some modifications in the attention domain, the syntactical and semantical complexity of language skill, and visuospatial ability items to accommodate removal of MMSE items. ACE-III assesses the domain of attention (18 points), memory (26 points), verbal fluency (14 points), language (26 points), and visuospatial (16 points) functions. ACE-III cognitive subscores correlate with the scores of comprehensive neuropsychological tests, and ACE-III has similar sensitivity and specificity for detecting Alzheimer's disease and frontotemporal dementia as ACE-R.

One of the most important differences is that the ACE-III does not provide the score of MMSE anymore. Therefore, a short form, the Mini-Addenbrooke's Cognitive Examination (Mini-ACE), was derived from ACE-III [36]. The administration time of ACE-III is 15–20 minutes, whereas Mini-ACE takes about 5 minutes to complete. Mini-ACE consists of only five items (attention: 4 points; memory: 7 points; verbal fluency: 7 points; clock drawing: 5 points; recall: 7 points) with a maximum score of 30. The Mini-ACE can be assessed as a stand-alone screening instrument or as a nested version in the ACE-III. Although several language validations of ACE-III and Mini-ACE exist [37–40], the diagnostic accuracy of ACE-III or Mini-ACE in the diagnosis of PD-MCI and PDD has not been investigated.

In the present study, we aimed to compare the sensitivity and specificity of the different ACE variants available in Hungarian (ACE-I, ACE-III, and Mini-ACE) for detecting mild and major NCD in PD in accordance with the DSM-5 criteria.

## 2. Materials and Methods

Five hundred and seventy-nine consecutive PD patients were recruited for the study. Patients were treated at the Department of Neurology, University of Pécs. Each patient fulfilled the clinical diagnostic criteria for PD [41] and gave written informed consent according to the approval of the Regional Ethical Board of the University of Pécs.

History of alcoholism, cerebrovascular disease, and other conditions known to impair mental status served as exclusion criteria for participation. Each patient had a routine brain MRI (or brain CT if the MRI examination was contraindicated). Patients with focal abnormalities on neuroimaging studies, abnormalities in thyroid hormone levels, or uncompensated systemic diseases (i.e., diabetes, hypertension, and heart failure) were also excluded. According to the abovementioned criteria, twenty-seven patients were excluded from the study.

Patients were evaluated using the Hungarian version of Mini-Mental State Examination (MMSE) [42], Addenbrooke Cognitive Examination I (ACE-I) [22] and III (ACE-III), Mini-Addenbrooke Cognitive Examination (Mini-ACE), Montgomery-Asberg Depression Rating Scale (MADRS) [43], and the Lille Apathy Rating Scale (LARS) [44, 45]. The severity of Parkinsonian symptoms was evaluated by the Hoehn-Yahr stage (HYS) [46], the Movement Disorders Society-sponsored Unified Parkinson's Disease Rating Scale (MDS-UPDRS) [47], and the Unified Dyskinesia Rating Scale (UDysRS) [48] if motor complications were present.

Subsequently, PD patients were divided into three groups based on the DSM-5 criteria: patients with major neurocognitive disorder (major NCD group), mild neurocognitive disorder (mild NCD group), and patients without any NCD (normal cognition) [24, 25].

Statistical analysis was performed using *IBM SPSS* (version 19, a SPSS Inc. IBM) and *R for Windows* 3.1.2 statistical software. Because data did not follow a normal distribution, Kruskal-Wallis nonparametric test was applied. Since HY and sex are dichotomous and categorical variables, Pearson's chi-square and Kendall-tau tests were applied to these variables. Receiver operating characteristic curve statistics for MMSE, ACE-I, ACE-III, and Mini-ACE were calculated in SPSS software.

## 3. Results

A total of 27 patients were excluded for the further analyses because of the presence of clinically severe depression and/or anxiety judged by the neuropsychological examination or abnormalities present on the neuroimaging. Out of 552 evaluated PD patients, 277 had normal cognition, 165 had mild NCD, and 110 had major NCD according to DSM-5 classification.  Table 1 shows the demographic and clinical characteristics of these groups. Except for levodopa equivalent dose (LED, mg) and sex, all demographic and clinical variables showed significant differences. There were significant differences in the total scores and the subscores of ACE-I, ACE-III, Mini-ACE, and MMSE between normal, mild, and major NCD groups.

Table 2 shows the results of the ROC analysis of detecting mild NCD based on the different levels of education years. According to Youden's index, ACE-III has the best diagnostic accuracy in individuals having 0–8 years of education (sensitivity: 93%; specificity: 64%, area under curve (AUC) area: 73%, cut-off score: 83.5 points) and 9–12 years of education (sensitivity: 80%, specificity: 78%, AUC area: 77%, cut-off score: 85.5 points). However, the Mini-ACE test has the best performance among subjects with >12 years of education (sensitivity: 71%, specificity: 79%, AUC area: 82%, cut-off score: 25.5 points).

For detecting major NCD (Table 3), ACE-III test has a best diagnostic accuracy across all levels of education. Among subjects with 0–8 years of education, its sensitivity and specificity was 80% and 90%, respectively (AUC: 89%, the cut-off score was 70.5 points). In PD patients with 9–12 years of formal education, the specificity and sensitivity of ACE-III was 90% and 74%, respectively (AUC area: 92% and the cut-off score: 77.5 points). In individuals having more than 12 years of education, its sensitivity and sensitivity was 95% and 92%, respectively (AUC: 97%, and the cut-off score: 78.5 points).

## 4. Discussion

Cognitive impairment influences the quality of life of Parkinson's patients and is detectable in newly diagnosed patients [6, 45, 49]. The diagnosis of PD-MCI is important because it could influence the therapeutic decisions and it also could be the harbinger of PDD [13, 50]. The frequency of PD-MCI and dementia in PD may be about 30% depending on the age, disease duration, and comorbidities. Based on its clinical relevance, it is critical to identify mild and major neurocognitive disorders in PD. Therefore, reliable, valid, and accurate screening tests are warranted in the clinical practice.

The three different variants of the Addenbrooke's Cognitive Examination may help establish the Level-I diagnosis [19]. The ACE test was originally developed for the discrimination of Alzheimer's disease from frontotemporal dementia [26]. Reyes et al. demonstrated 92% sensitivity and 91% specificity of ACE-I (cut-off score: 83) in the detection of dementia of a small number of patients with Parkinson's disease [51]. In a small group of Hungarian subjects with Parkinson's disease, the ACE-I sensitivity and specificity in detecting dementia were 74% and 78.1% with 80 point cut-off score of [22]. Lucza et al. demonstrated 83.5 point cut-off score of ACE-I (sensitivity: 87.1%, specificity: 79.7%) in differentiating mild neurocognitive disorder in PD, and 80.5 points (sensitivity: 86.9%, specificity: 73.7%) in PD with major neurocognitive disorder [24, 25].

The next version of the Addenbrooke's test family, the ACE-R, has never been formally translated and validated in Hungary. Therefore, in this study, we could not evaluate its discriminative capabilities. However, it has been utilized internationally for screening PD-MCI and PDD. The sensitivity and specificity of ACE-R detecting PD-MCI are 69% and 84% with 89 point cut-off score of [52]. ACE-R was also used to differentiate cognitive functions in PD from Parkinsonian syndromes [53]. This study demonstrated the usefulness of verbal fluency subscore in the differential diagnosis of PDD from Parkinsonism. ACE-R has 85.5 point cut-off score (sensitivity: 68%, specificity: 91%) in discriminating PD-MCI from PD with normal cognition and 82.5 points (sensitivity: 70%, specificity: 73%) in discriminating PD-MCI from PDD in Czech PD population (a sample of 69 persons) [54].

The newest versions of the ACE test batteries, the ACE-III and Mini-ACE, have never been tested previously for the detection of mild and major NCD in PD (PubMed search using the keywords “Parkinson's disease” AND “ACE-III” OR “Parkinson's disease” AND “ACE III”, performed on July 29, 2018). In our cohort of patients, ACE-III had better discriminative abilities than ACE-I had. Moreover, ACE-III was also better in screening for mild and major NCD than the MMSE. Generally, the discriminative accuracy of Mini-ACE was between those of ACE and ACE-III with the exception of screening for mild NCD in PD among subjects having > 12 years of formal education.

As far as the authors are aware of, this is the first validation study of ACE-III and Mini-ACE for detecting mild and major NCDs in PD. In the present study, we demonstrated that ACE has an acceptable sensitivity for detecting both mild and major NCD; however, its specificity was below the expectation in the group of patients having low education levels (up to and including 8 years of education). One of the strengths of our study was that we could establish valid and reliable discriminative thresholds for different education levels. Although we have included a large pool of patients with wide age distribution and various disease severity stages to improve the consistency of our data, we are also aware of some limitations. Despite of measuring the MADRS, we did not use these scores for excluding patients with depression. Because the symptoms of depression are largely overlapping with numerous features of PD (e.g., slowness of thinking, sexual disturbances, and changes in appetite) and many subjects experience nonmotor fluctuations (e.g., having depressed mood or anxiety in OFF state while not having such phenomena in the ON periods), they might have higher MADRS scores without clinically significant depression. Therefore, we decided to exclude only those patients who had severe depression diagnosed by the neuropsychologists (*n* = 27) but not based on the MADRS values alone.

## Figures and Tables

**Table 1:** Demographic and clinical characteristics of Parkinson's disease patients with normal cognition, mild and major neurocognitive disorder.

	Normal cognition (*N* = 277)					Mild neurocognitive disorder (*N* = 165)					Major neurocognitive disorder (*N* = 110)					
	Median	Percentile 25	Percentile 75	Mean	Standard deviation	Median	Percentile 25	Percentile 75	Mean	Standard deviation	Median	Percentile 25	Percentile 75	Mean	Standard deviation	*p* value
Age	64	56	68	61.80	9.80	69	65	74	68.81	7.73	73	66	78	71.84	7.70	0.000
Education	13	11	16	13.63	2.98	12	11	13	11.97	2.85	11	8	12	11.01	3.39	0.000
Sex (M/F)^a^	157/120					94/71					66/44					0.82
Disease onset	57	48	64	55.36	11.55	61	53	69	61.21	10.73	65	56	74	63.73	12.22	0.000
Disease duration	5	2	9	6.36	6.04	6	2	11	7.57	6.28	6	2	13	8.06	7.06	0.04
MDS UPDRS part1	10	6	15	10.76	6.33	14	9	19	14.44	6.77	19	13	24	18.97	6.65	0.000
MDS UPDRS part2	10	5	17	11.17	7.60	14	9	20	14.75	7.72	20	12	27	19.61	8.82	0.000
MDS UPDRS part3	26	18	35	27.97	13.20	35	27	42	35.63	12.82	47	39	56	48.13	13.45	0.000
MDS UPDRS part4	4	1	5	3.89	3.20	4	2	6	4.30	3.33	4	4	6	5.11	3.27	0.003
Hoehn-Yahr stage^a^	2	2	2	2.25	.72	2	2	3	2.62	.85	3	3	4	3.27	.86	0.000
LED (mg)	480	150	920	616.29	582.09	680	200	1020	732.89	621.08	522	150	950	639.25	556.31	0.091
MADRS total score	8	4	13	8.8	6.3	12	7	17	12.7	6.9	17	12	20	17.0	6.6	0.000
MMSE total score	29	28	30	28.72	1.32	28	26	29	27.41	1.76	25	22	27	24.20	3.12	0.000
ACE-I total score	92	87	95	90.31	5.91	83	77	87	82.26	6.83	69	63	75	68.55	8.79	0.000
ACE-I orientation	10	10	10	9.92	.32	10	10	10	9.76	.63	10	9	10	9.05	1.46	0.000
ACE-I attention	8	8	8	7.83	.53	8	7	8	7.48	.95	7	5	8	6.33	1.73	0.000
ACE-I memory	31	28	33	30.33	4.05	27	22	30	25.96	4.99	19	16	24	19.62	5.39	0.000
ACE-I verbal fluency	11	9	12	10.39	2.05	9	7	10	8.38	2.34	6	4	7	5.53	2.41	0.000
ACE-I language	28	27	28	27.53	.76	27	27	28	27.04	1.11	26	25	27	25.65	1.94	0.000
ACE-I spatial visual	5	4	5	4.30	.90	4	3	5	3.65	1.13	2	1	3	2.37	1.31	0.000
ACE-III total score	91	86	95	90.08	6.00	83	77	87	81.92	6.48	68	60	76	66.99	9.91	0.000
ACE-III attention	18	17	18	17.37	1.02	17	16	18	16.63	1.47	14	13	17	14.55	2.46	0.000
ACE-III memory	23	21	25	22.56	3.15	20	17	22	19.44	3.99	14	12	18	14.45	4.53	0.000
ACE-III verbal fluency	11	9	12	10.36	2.09	9	7	10	8.42	2.31	5	4	7	5.40	2.44	0.000
ACE-III language	26	25	26	25.09	1.42	24	23	25	23.88	1.96	22	20	24	21.58	3.08	0.000
ACE-III spatial visual	15	14	16	14.69	1.52	14	12	15	13.55	1.88	11	9	13	11.01	2.73	0.000
Mini-ACE total score	27	25	29	26.4	2.9	23	20	25	22.4	3.4	16	13	19	15.8	4.3	0.000

ACE-I: Addenbrooke's Cognitive Examination version I; ACE-III: Addenbrooke's Cognitive Examination version III; Mini-ACE: Mini Addenbrooke's Cognitive Examination; MMSE: Mini Mental State Examination; MDS UPDRS: Movement Disorders Society Unified Parkinson's Disease Rating Scale (Pearson chi-square and Kendall-tau).

**Table 2:** Diagnostic accuracy of ACE-I, ACE-III, Mini-ACE, and MMSE for detecting mild neurocognitive disorder in the respect of education years.

Mild neurocognitive disorder		Best cut-off score	Sensitivity	Specificity	Youden's index	Positive likelihood ratio	Negative likelihood ratio	AUC area
ACE-I	0–8	84.5	0.862	0.571	0.433	2.011	0.241	0.713
	9–12	86.5	0.850	0.614	0.464	2.204	0.244	0.787
	>12	90.5	0.847	0.626	0.473	2.263	0.244	0.819

ACE-III	0–8	83.5	0.931	0.643	0.574	2.607	0.107	0.733
	9–12	85.5	0.805	0.786	0.511	2.917	0.477	0.771
	>12	88.5	0.765	0.744	0.509	2.985	0.316	0.838

Mini-ACE	0–8	23.5	0.793	0.714	0.507	2.776	0.290	0.749
	9–12	24.5	0.850	0.514	0.364	1.750	0.292	0.746
	>12	25.5	0.718	0.798	0.516	3.553	0.354	0.820

MMSE	0–8	26.5	0.414	0.857	0.271	2.897	0.684	0.602
	9–12	27.5	0.575	0.743	0.318	2.236	0.572	0.723
	>12	28.5	0.612	0.714	0.326	2.141	0.544	0.696

**Table 3:** Diagnostic accuracy of ACE-I, ACE-III, Mini-ACE, and MMSE for detecting major neurocognitive disorder in the respect of education years.

Major neurocognitive disorder		Best cut-off score	Sensitivity	Specificity	Youden's index	Positive likelihood ratio	Negative likelihood ratio	AUC area
ACE-I	0–8	74.5	0.821	0.744	0.565	3.207	0.241	0.871
	9–12	76.5	0.848	0.773	0.622	3.743	0.196	0.915
	>12	80.5	0.903	0.927	0.881	13.076	0.050	0.974

ACE-III	0–8	70.5	0.805	0.907	0.702	8.545	0.226	0.895
	9–12	77.5	0.909	0.740	0.649	3.497	0.123	0.926
	>12	78.5	0.957	0.927	0.894	12.439	0.100	0.972

Mini-ACE	0–8	17.5	0.718	0.791	0.509	3.430	0.357	0.833
	9–12	20.5	0.833	0.747	0.580	3.289	0.223	0.894
	>12	21.5	0.907	0.917	0.824	10.884	0.101	0.968

MMSE	0–8	25.5	0.744	0.860	0.604	5.329	0.298	0.866
	9–12	26.5	0.803	0.780	0.583	3.650	0.253	0.868
	>12	27.5	0.814	0.809	0.623	4.262	0.230	0.903

## Data Availability

The data used to support the findings of this study have not been made available because the current ethical approval does not permit its deposition.

## Abbreviations

ACE: Addenbrooke's Cognitive Examination
AUC: Area under the curve
Mini-ACE: Mini Addenbrooke's Cognitive Examination
MMSE: Mini-Mental State Examination
MADRS: Montgomery-Asberg Depression Rating Scale
DSM-5: Diagnostic and Statistical Manual of Mental Disorders 5th edition
LED: Levodopa equivalent dosage
MCI: Mild cognitive impairment
NCD: Neurocognitive disorder
ROC: Receiver operating characteristic
HYS: Hoehn-Yahr stage
MDS-UPDRS: Movement Disorders Society Sponsored version of Unified Parkinson's Disease Rating Scale
PD: Parkinson's disease
PD-MCI: Mild cognitive impairment in Parkinson's disease.
